# Anxiety and Startle Phenotypes in *Glrb Spastic* and *Glra1 Spasmodic* Mouse Mutants

**DOI:** 10.3389/fnmol.2020.00152

**Published:** 2020-08-11

**Authors:** Natascha Schaefer, Jérémy Signoret-Genest, Cora R. von Collenberg, Britta Wachter, Jürgen Deckert, Philip Tovote, Robert Blum, Carmen Villmann

**Affiliations:** ^1^Institute of Clinical Neurobiology, University Hospital, Julius Maximilians University of Würzburg, Würzburg, Germany; ^2^Department of Psychiatry, Psychosomatics and Psychotherapy, Center for Mental Health, University Hospital Würzburg, Würzburg, Germany

**Keywords:** glycine receptor, spastic, fear, anxiety, startle reaction

## Abstract

A GWAS study recently demonstrated single nucleotide polymorphisms (SNPs) in the human *GLRB* gene of individuals with a prevalence for agoraphobia. *GLRB* encodes the glycine receptor (GlyRs) β subunit. The identified SNPs are localized within the gene flanking regions (3′ and 5′ UTRs) and intronic regions. It was suggested that these nucleotide polymorphisms modify GlyRs expression and phenotypic behavior in humans contributing to an anxiety phenotype as a mild form of hyperekplexia. Hyperekplexia is a human neuromotor disorder with massive startle phenotypes due to mutations in genes encoding GlyRs subunits. *GLRA1* mutations have been more commonly observed than *GLRB* mutations. If an anxiety phenotype contributes to the hyperekplexia disease pattern has not been investigated yet. Here, we compared two mouse models harboring either a mutation in the murine *Glra1* or *Glrb* gene with regard to anxiety and startle phenotypes. Homozygous *spasmodic* animals carrying a *Glra1* point mutation (alanine 52 to serine) displayed abnormally enhanced startle responses. Moreover, *spasmodic* mice exhibited significant changes in fear-related behaviors (freezing, rearing and time spent on back) analyzed during the startle paradigm, even in a neutral context. *Spastic* mice exhibit reduced expression levels of the full-length GlyRs β subunit due to aberrant splicing of the *Glrb* gene. Heterozygous animals appear normal without an obvious behavioral phenotype and thus might reflect the human situation analyzed in the GWAS study on agoraphobia and startle. In contrast to *spasmodic* mice, heterozygous *spastic* animals revealed no startle phenotype in a neutral as well as a conditioning context. Other mechanisms such as a modulatory function of the GlyRs β subunit within glycinergic circuits in neuronal networks important for fear and fear-related behavior may exist. Possibly, in human additional changes in fear and fear-related circuits either due to gene-gene interactions e.g., with *GLRA1* genes or epigenetic factors are necessary to create the agoraphobia and in particular the startle phenotype.

## Introduction

Disturbances of glycinergic inhibition in the central nervous system have been associated with neuromotor disorders, changes in pain sensation and autism (Harvey et al., 2004; Chung et al., 2010; Pilorge et al., 2016).

Glycine receptors (GlyRs) enable fast synaptic inhibition in the adult spinal cord and brain stem. They are important for motor coordination and respiratory rhythm (Manzke et al., 2010). Together with the GABA_A/C_ receptor, the 5HT_3_ receptors, and nicotinic acetylcholine receptors (nAChRs), GlyRs are members of the superfamily of Cys-loop receptors (CLRs) (Miller and Smart, 2010). Four possible isoforms of GlyR α-subunits are described (α1 to α4), but only one isoform of the β-subunit. The adult synaptic receptor complex in brainstem and spinal cord is composed of 3α:2β subunits (either α1β or α3β) (Durisic et al., 2012; Patrizio et al., 2017). GlyR β has been also detected outside the brainstem and spinal cord e.g., within the cortex, the habenular nucleus of the hypothalamus and the interposed and medial nuclei of the cerebellum suggesting that the β subunit may have other so far unknown functions (Kingsmore et al., 1994; Weltzien et al., 2012).

Numerous studies investigating disturbances in glycinergic inhibition rely on mutations within the receptor genes *GLRA1* and *GLRB* which have been associated with startle disease (hyperekplexia) derogating the nerve-muscle circuit (Schaefer et al., 2012; Chung et al., 2013). Human hyperekplexia patients suffer from exaggerated startle responses, enhanced muscle tone, and stiffness in infancy.

Glycinergic mouse models have been used to study pathomechanisms of startle disease. *Oscillator*, *spasmodic*, and *shaky* mice harbor *Glra1* mutations. The currently only available mouse model carrying a *Glrb* mutation is the *spastic* mouse. *Oscillator* represents a functional *Glra1* NULL mutation due to a microdeletion. *Shaky* and *spasmodic* carry missense mutations in the N-terminus of the GlyR α1 subunit (Schaefer et al., 2012). *Spasmodic* mice harbor a point mutation (A52S) within the GlyR α1 subunit. The A52S mutation reduces the affinity of the neurotransmitter glycine to the GlyRs and at the functional level alters the efficacy of receptor activation (Saul et al., 1994; Plested et al., 2007). *Spasmodic* mice are known to display changes in startle behavior (Plappert et al., 2001). Homozygous *spastic* mice also develop massive startle reactions typical for startle disease (Kingsmore et al., 1994; Becker et al., 2012). The underlying mutation is an insertion of a LINE-1 element into intron 6 of the *Glrb* gene resulting in aberrant splicing and therefore lower expression levels (<25%) of the full-length GlyRs β subunit in homozygous *Glrb*^*spa/spa*^ mice (Kingsmore et al., 1994). Heterozygous *Glrb^+/spa^* mice, however, do not show any deficits in motor behavior along with preservation of 70–80% of α1/β receptors (Becker et al., 1986; Hartenstein et al., 1996). At functional level, heterozygous *spastic* mice have been investigated electrophysiologically in superficial dorsal horn (SFDH) neurons. The analysis of mIPSCs revealed no changes in rise time, decay time and frequency compared to wild type mice. In contrast, a dramatic change in channel characteristics leading to decreased glycinergic inhibition in SFDH neurons was described for homozygous *spastic* mice (Graham et al., 2003). Current knowledge on heterozygous *spastic* mice indicates reduced GlyR β protein levels as the major consequence of the *Glrb* mutation.

In the human situation, it has been reported that affected patients with startle disease are anxious to fall due to unexpected noise or tactile stimuli (Andermann et al., 1980). Recently, a link of GlyRs to agoraphobia (Deckert et al., 2017) has been suggested. Agoraphobia is a DSM-V or ICD-10 diagnosis assigned to subjects who suffer from disproportionate fear of public places, often perceiving such environments as too open, crowded or dangerous (Wittchen et al., 2010). A recent GWAS study used an Agoraphobic Cognition Questionnaire (ACQ) and identified several allelic variations within the human *GLRB* gene (intronic region and flanking regions 3′ and 5′ UTRs) suggesting *GLRB* as a candidate gene for agoraphobia (Deckert et al., 2017). Family/twin studies have shown familial aggregation in agoraphobia with an estimated heritability of 48% percent for agoraphobia arguing for an inherited component (Mosing et al., 2009). In a family with hyperekplexia, Kirstein and Silfverskiold provided the first evidence that seizures or startle attacks can be “emotionally elicited, i.e., evoked by surprise, fear, or stress” (Kirstein and Silfverskiold, 1958).

Here we comprehensively characterized anxiety-like and startle behavior in male heterozygous *spastic* as well as homozygous *spasmodic* mice. Homozygous *spasmodic* mice displayed significantly decreased grooming and rearing but increased freezing and startle-induced seizure-like motor episodes whereas *spastic* mice were inconspicuous in diverse paradigms measuring startle behavior.

## Materials and Methods

### Animals

Adult *Glra1*^*spd*^ mice (*spasmodic*, JAX stock #001278) and *Glrb*^*spa*^ mice (*spastic*, JAX stock #000241) both from Jackson Laboratories, Bar Harbor, ME, United States were used (Kingsmore et al., 1994; Ryan et al., 1994). Mice of both lines were kindly provided by Cord-Michael Becker (FAU Erlangen-Nürnberg, Germany) and transferred into the animal facility of the Institute of Clinical Neurobiology (Würzburg, Germany). Here, *spastic* mice were backcrossed to C57BL/6J background for at least 15 generations. *Spasmodic* mice have a C3H background and were backcrossed for more than 15 generations. All experiments were in accordance with European Union guidelines and approved by our local veterinary authority (Veterinäramt der Stadt Würzburg) and Committee on the Ethics of Animal Experiments (Regierung von Unterfranken, Würzburg). The experiments were authorized under reference numbers 55.2-2531.01-09/14; 55.2.2-2532.2-536.

For behavioral investigation, male mice (wild type animals *Glra1^+/+^* and homozygous mutants *Glra1*^*spd/spd*^ and *Glrb^+/+^* and *Glrb^+/spa^* animals always from same litters) were transferred to the behavioral unit at an age of 10–11 weeks where they were kept for 1 week prior to experiments until the end of the study. Male mice were housed individually with access to water and food *ad libitum* at a 12 h light/dark rhythm with lights on at 6.30 a.m. Behavioral studies were performed between 7 a.m. and 6 p.m. during the light cycle. Bodyweight was checked 1 day prior to each behavioral experiment.

### Mouse Genotyping

Genotyping of mice was performed from an ear punch at the age of 3 weeks. Genotypes of *spastic* mice were defined via PCR before and after experimental procedure: *Glrb^+/spa^* produce a 250 and 200 bp product with primer pairs AIN/SIN and AIN/SPA (AIN: AACACAGAGCAATTATATTTTAGAAG; SIN: AAGAAGACAGAGCTTTCCATTGT; SPA: AATTTCCTA AGTTCCGGT) in a standard PCR protocol. Wild type mice from the same litter gave rise to only 250 bp product combining AIN with SIN.

Genotypes of *spasmodic* mice were defined via PCR before and after experimental procedure. *Glra1^+/spd^* produce a 194 and 187 bp product with primer pairs N/rev and M/rev (N: TACTCACCTATGGTTGTCTCAGC; M: TACTCACCTATGGT TGTCTCAGA; rev: TAGTCTGGCAGAGATGCTAAATG) in a standard PCR protocol. In wild type control mice of the same litter, the PCR gave rise to a 194 bp product, in homozygous *Glra1*^*spd/spd*^ only a 187 bp fragment was obtained.

### Rotarod

Motor coordination of mice was tested using a rotarod (accelerating rotarod, Ugo Basile). The latency until mice fell off the rod or started clinging to the rod was recorded. Nine wild type *Glrb^+/+^* animals and 10 *Glrb^+/spa^* were tested at 1 day in three trials with a 2 h break in between. Each trial consisted of two runs. Starting speed was 5 rpm and accelerated every 30 s until 50 rpm. Total duration was 4.5 min.

### Open Field

Wild type mice *Glra1^+/+^* and homozygous *Glra1*^*spd/spd*^ mice were placed in a 48 × 48 cm square box, illuminated with ∼40 lux. Animals were monitored for 5 min each and tracked with the Video Mot Software (TSE Systems, Bad Homburg, Germany). For analysis, the box was divided into fields of interest: center of the arena (24 × 24 cm) versus the periphery. Entries into the center, time spent in the center and traveled distances were recorded using a one-point body tracking.

### Elevated Plus Maze

The elevated plus maze (EPM) made of white frosted plastic was used to assess anxiety-like behavior by analyzing the activity in open versus closed arms (Walf and Frye, 2007). The EPM (TSE Systems, Bad Homburg, Germany; length of arm: 30 cm, width: 5 cm, height of closed arm: 15 cm, height above ground: 48 cm) consisted of two closed and two open arms. Luminosity was adjusted to ∼ 60 lux. Mice were video tracked for 10 min using the Video Mot Software (TSE Systems, Bad Homburg, Germany, camera: Logitech). The following parameters were analyzed and compared between open and closed arms: distance traveled and time spent in open and closed arms for the first 5 min.

### Dark/Light Test

Mice were subjected to a dark/light transition test to determine the innate aversion of rodents to brightly illuminated areas and their spontaneous exploratory behavior. The dark-light transition test was performed in the open field (OF) arena. A red acrylic glass box of 47 × 16 × 25 cm was positioned in the box. The dark compartment box carried a small entrance and covered one third of the box. The rest of the box was brightly illuminated. Mouse movements were tracked for 10 min using the Video Mot Software (TSE Germany, camera: Logitech). The following parameters were recorded and analyzed: distance traveled in the light compartment and time spent in each compartment.

### Hot Plate

The hot plate setup (custom made) consisted of a viewing jar, a hollow acrylic glass cylinder (18.7 cm high and 14.2 cm wide) placed on a hot plate (IKA, RCT basic, Staufen, Germany). The temperature was regulated and monitored with a thermometer (IKA, ETS-D5, Staufen, Germany) connected to the metal block. Mice were placed in the viewing jar with the metal block heated up to 54°C (±1°C). The time until the mice licked their hind paws was measured, and the mice were immediately removed from the plate. If the mice started jumping or vocalizing, they were immediately removed from the plate. If none of these criteria were applicable mice were taken off the hot plate after 30 s to prevent tissue damage or other injuries.

### Startle Behavior

A motion sensitive platform with three piezoelectric sensors that transduced the animal’s motion into a voltage signal was enclosed by a polycarbonate mouse cage separated into two non-contacting sections held by an aluminum frame. The motion-sensitive platform was custom-made as described in Daldrup et al. (2015). The lower section of the mouse cage (length: 25 cm, width: 19 cm, height: 10.5 cm) was suspended ∼0.5 cm above the motion sensitive platform, and the upper section (25 × 19 × 11.5 cm) had an LED port attached to the outer part in order to signal the triggering of the white noise. The startle apparatus was located inside a sound attenuated box (100 × 80 × 116 cm) and dimly lit from above by a circular LED lamp. The piezoelectric voltage signal was continuously recorded at a 5 kHz sampling rate. The startle stimuli consisted of wide band white noise bursts (20 ms duration) generated by an RZ6 multi-processor and delivered via a multi-field magnetic speaker (MF1) located 20 cm above the motion sensitive platform. The triggering of the startle stimuli was controlled by a Real-time Processor Visual Design Studio software (RPvdsEx; RZ6, MF1 and RPvdsEx are from Tucker-Davis Technologies, Alachua, FL, United States).

A camera located on the side of the cage controlled by video tracking software (CinePlex Studio; Plexon Inc., Dallas, TX, United States) was used to record the animal’s behavior during the startle experiments. Mice were acclimated to the context for 300 s, during which no sound was presented. This was immediately followed by 20 startle-eliciting white noise bursts, presented with a randomized inter-stimulus interval of 30–50 s. The 20 bursts were presented in five series, each containing four different intensities (70, 80, 90, and 100 dB) in a pseudorandomized order. Total duration of startle paradigm was 1110 s.

The startle amplitude was defined as the maximum amplitude in the voltage signal occurring within a 100 ms time window after the onset of each white noise burst, whose threshold was above the mean ± 2 standard deviations of a 500 ms pre-stimulus voltage baseline.

### Fear Conditioning

Mice were subjected to auditory fear conditioning in a brightly illuminated square context (27 cm × 27 cm) with a metal grid floor. A train of 20 tone beeps (7.5 kHz, 75 dB sound pressure level, 500 ms duration, 500 ms inter-beep-interval) was used as the conditioned stimulus (CS) and an electrical foot-shock (0.7 mA dc = direct current, 1 s duration) was used as the unconditioned stimulus (US). During the conditioning session, the mice were exposed to three back-to-back CS–US pairings with a baseline period of 180 s and a minimal inter-stimulus interval of 80 s. On the day after conditioning, mice were exposed to four CS-only presentations in a dimly illuminated context different from the conditioning context after a baseline period of 180 s. While the conditioning context was cleaned with 70% ethanol, the retrieval context was wiped down with 1% acetic acid.

Sounds and shocks presentations were controlled by the same system as for the startle experiments, and movies were recorded from top with the Plexon Cineplex software. The animal’s motion was computed from the video recordings by determining the pixel change across frames with custom written MATLAB (MathWorks) code. Freezing episodes were defined as periods when the motion was below an absolute threshold value (the same was used for all the animals). Only events longer than 2 s were defined as freezing episodes, and events closer than 200 ms were merged.

### Statistical Analysis

Data are represented as mean ± S.E.M. (standard error of the mean) and were analyzed using Graph Pad Prism or Origin 9 Software. Differences between genotypes were tested using unpaired *t*-test except for analysis of the startle data where a repetitive ANOVA (two-way ANOVA) with Bonferroni *post hoc* test was performed. The 0-hypothesis was rejected at a level of *p* < 0.05.

## Results

### Heterozygous *Glrb^+/spa^* Mice Exhibit Normal Motor Behavior and No Change in Pain Sensation Compared to Wild Type Control Animals

Previous studies linked the GlyR β subunit to fear and anxiety-like behavior (Deckert et al., 2017; Lueken et al., 2017). Deckert et al. (2017) demonstrated in humans with *GLRB* polymorphisms an increase in startle responses. Likewise, heterozygous *spastic* mice spent less time in the center than in the periphery of an open field compared to wild type control animals.

**FIGURE 1:**
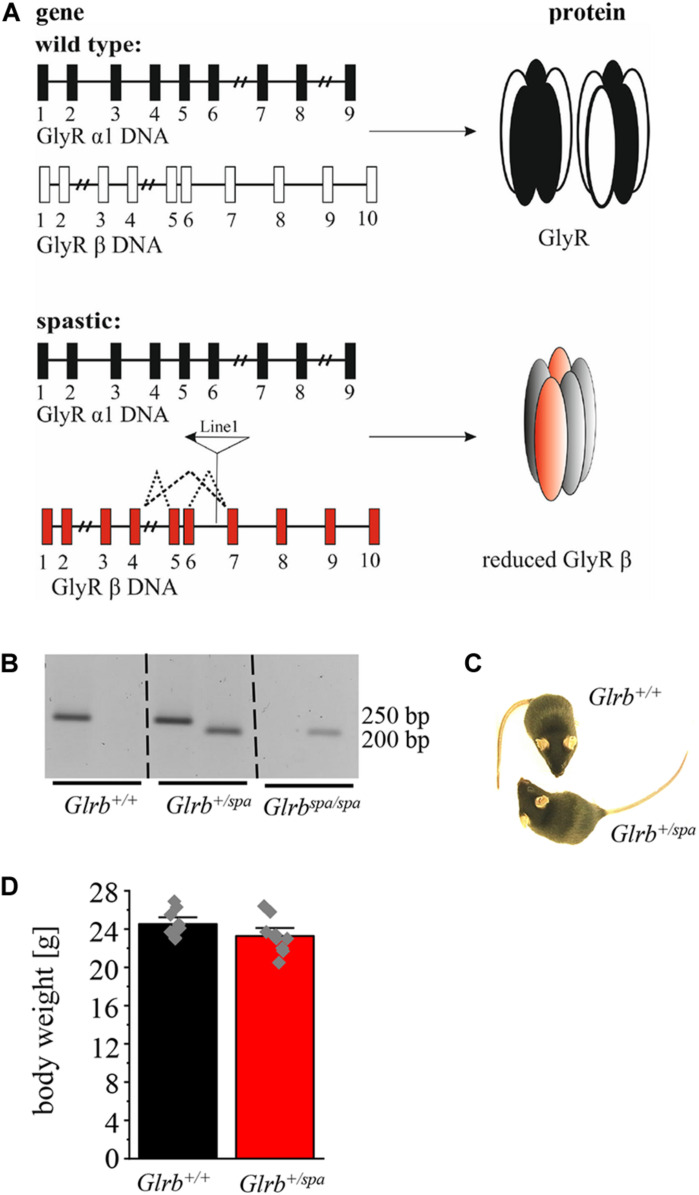
A LINE-1 insertion into the *Glrb* gene underlies the mouse mutant *spastic*. **(A)** Schematic overview of *Glra1* (9 exons) and *Glrb* (10 exons) genes and protein level. Upper part shows the wild type situation (black and white), lower part depicts the situation in *spastic* mice (black and red). Left column depicts changes at DNA levels, see insertion of LINE-1 element in *Glrb* generating *spastic* mice (red exons) leading to different splice variants indicated by dotted lines. Right column: protein level of GlyRs. Note that the amount of the full length GlyR β is reduced in *spastic* mice. **(B)** Representative image of genotyped mice (*Glrb^+/+^*, *Glrb^+/spa^*, and *Glrb*^*spa/spa*^ mice). Wild type (wt) animals show a single 250 bp band, heterozygous animals show two bands (250 and 200 bp), homozygous *spastic* mice display a band of 200 bp. **(C)** Images of wt *Glrb^+/+^* and heterozygous *spastic Glrb^+/spa^* animals with no obvious changes in size, and appearance of the coat. **(D)** Comparison of the body weight of *Glrb^+/spa^* mice (*n* = 10, red bar) and *Glrb^+/+^* mice (*n* = 9, black bar) investigated in this study. Body weight data show mean values ± standard error of the mean (S.E.M). Single data points are shown as gray color squares.

In the present study, heterozygous *spastic* mice were investigated to further elucidate the impact of the mutant GlyR β subunit gene on anxiety-like behavior. *Spastic* mice harbor a LINE-1 insertion within intron 6 of the *Glrb* gene (Figure 1A) leading to aberrant splice variants of the GlyR β subunit resulting in reduced full-length protein (Becker et al., 2012). Following genotyping of *spastic* mice (Figure 1B), the phenotypic appearance of *Glrb^+/spa^* mice compared to *Glrb^+/+^* wild type (wt) littermates was investigated. No differences in the outer appearance like quality of fur (color or thickness) was observed (Figure 1C). In addition, no differences in body weight were detected (*Glrb^+/spa^ n* = 10, 23.3 ± 0.6 g; *Glrb^+/+^ n* = 9, 24.5 ± 0.5 g; *p* = 0.11, *t* = 1.697, df = 17, *F*-value = 1.608) (Figure 1D).

**FIGURE 2:**
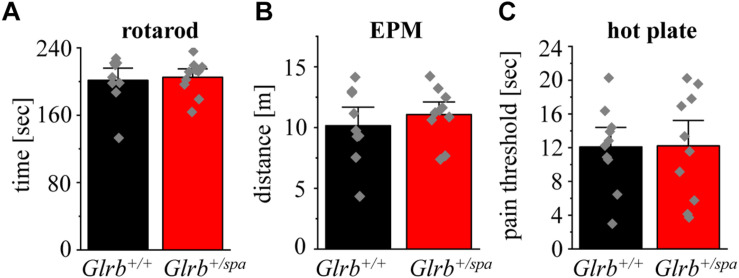
Motor behavior and pain sensation are unaffected in heterozygous *spastic* animals. **(A)** Rotarod performance of *Glrb^+/spa^* mice (*n* = 10, red bar) and *Glrb^+/+^* mice (*n* = 9, black bar) with accelerating speed. Time until mice fall off the rod is shown as mean. **(B)** Traveled distance of *Glrb^+/spa^* mice (*n* = 10, red bar) and *Glrb^+/+^* mice (*n* = 9, black bar) within open and closed arms in the elevated plus maze (EPM) test is depicted. **(C)** *Glrb^+/spa^* mice (*n* = 10, red bar) and *Glrb^+/+^* mice (*n* = 10, black bar) were placed on a hot plate. Threshold until mice licking their hind paws was detected. Error bars refer to S.E.M. values. Data points are shown as gray color squares.

As impaired motor behavior interferes with the readout of anxiety tests, we further analyzed motor coordination in wild type animals versus heterozygous *spastic* littermates. Performing accelerating rotarod test (Figure 2A), heterozygous *spastic* mice did not show differences in time spent on the rod (*n* = 10, 205.1 ± 6.6 s) in comparison to wild type littermates (*n* = 9, 201.4 ± 9.7 s; *p* = 0.75, *t* = 0.32, df = 17, *F*-value = 1.945). In the EPM paradigm, no significant changes were observed in the distance traveled (*Glrb^+/spa^ n* = 10, 11 ± 2.2 m; *Glrb^+/+^ n* = 9, 10 ± 1.0 m; *p* = 0.45, *t* = 0.76, df = 17, *F*-value = 1.983) (Figure 2 B). The hot plate test investigates the thermal nociception of rodents (Allen and Yaksh, 2004). The test was used to exclude changes in perception of heterozygous *spastic* mice due to changed *Glrb* levels. Wild type and heterozygous *spastic* mice stayed 12 s on the hot plate (*Glrb^+/spa^ n* = 10, 12.2 ± 0.5 s; *Glrb^+/+^ n* = 10, 12.1 ± 1.4 s; *p* = 0.96, *t* = 0.05, df = 18, *F*-value = 1.7) (Figure 2C). We conclude that *Glrb^+/spa^* animals fulfill the prerequisites such as unchanged motor tasks to further study fear-related behavior.

### Heterozygous *Spastic* Mice Reveal Only Slight Preferences for the Dark Compartment

To investigate the impact of *Glrb* levels on fear-related behavior, heterozygous *Glrb^+/spa^* mice were monitored in the EPM and in the dark/light test and compared to wild type control littermates.

**FIGURE 3:**
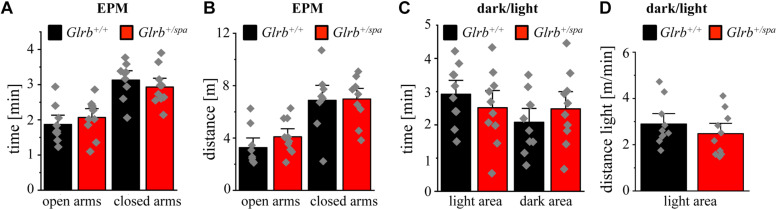
Heterozygous *spastic* mice prefer protected areas. **(A)** Time spent and **(B)** distance traveled in closed or open arms in the EPM for *Glrb^+/spa^* mice (*n* = 10, red bars) and *Glrb^+/+^* mice (*n* = 9, black bars). **(C)** Times spent within the dark or light compartments are detected for *Glrb^+/spa^* mice (*n* = 10, red bars) and *Glrb^+/+^* mice (*n* = 10, black bars). **(D)** Distance traveled for *Glrb^+/spa^* mice (*n* = 10, red bar) and *Glrb^+/+^* mice (*n* = 10, black bar) within the light compartment divided by time spent within the light compartment is depicted as bar diagram. Data are presented as mean values ± S.E.M. Data points are shown as gray color squares.

No differences in the time spent in open and closed arms were detected between heterozygous *spastic* mice and control animals (closed arms: *Glrb^+/spa^ n* = 10, 2.9 ± 0.2 min; *Glrb^+/+^ n* = 9, 3.1 ± 0.2 min; *p* = 0.42, *t* = 0.817, df = 17, *F*-value = 1.0; open arms: *Glrb^+/spa^ n* = 10, 2.1 ± 0.2 min; *Glrb^+/+^ n* = 9, 1.8 ± 0.2 min; *p* = 0.42, *t* = 0.817, df = 17, *F*-value = 1.0). Both, wild type and heterozygous *spastic* animals preferred the closed arms compared to the open arms (Figure 3A). In line with the total distance traveled (Figure 2B), the distances walked in the open and the closed arms were unaltered between heterozygous *spastic* mice and wild type littermates (closed arms: *Glrb^+/spa^ n* = 10, 7.0 ± 0.5 m; *Glrb^+/+^ n* = 9, 6.9 ± 0.8 m; *p* = 0.91, *t* = 0.102, df = 17, *F*-value = 1.783; open arms: *Glrb^+/spa^ n* = 10, 2.1 ± 0.2 m; *Glrb^+/+^ n* = 9, 1.9 ± 0.2 m; *p* = 0.21, *t* = 1.299, df = 17, *F*-value = 1.275, Figure 3B). To further examine anxiety-related behavior, the dark/light test was performed. Heterozygous *Glrb^+/spa^* mice were investigated for the time spent in the light compartment or the dark compartment of the arena. Wild type littermates were expected to explore the light compartment, spending more time in the light than in the dark area. Indeed, *Glrb^+/+^* mice spent significantly more time in the light than in the dark compartment (light compartment: *Glrb^+/+^ n* = 10, 2.9 ± 0.3 min; dark compartment: *Glrb^+/+^ n* = 10, 2.1 ± 0.3 min; *p* = 0.046, *t* = 2.139, df = 18, *F*-value = 1.0) whereas heterozygous *Glrb^+/spa^* spent the same amount of time within dark and light compartments (dark compartment: *Glrb^+/spa^ n* = 10, 2.5 ± 0.3 min; *Glrb^+/+^ n* = 10, 2.1 ± 0.3 min; *p* = 0.37, *t* = 0.9132, df = 18, *F*-value = 1.526; light compartment: *Glrb^+/spa^ n* = 10, 2.5 ± 0.3 min; *Glrb^+/+^ n* = 10, 2.9 ± 0.3 min; *p* = 0.37, *t* = 0.9132, df = 18, *F*-value = 1.526). The slightly increased time spent in the dark arena did not, however, reach significance (Figure 3C). Mice were also monitored for the distance traveled within the light compartment. The determination of the distance traveled in the dark compartment was impossible due to experimental setup. As the distance depends on the time spent within the light compartment, the traveled distance divided by time was analyzed. Again, no obvious differences in distance traveled were observed between both genotypes (*Glrb^+/spa^ n* = 10, 2.5 ± 0.3 m/min; *Glrb^+/+^ n* = 9, 2.9 ± 0.3 m/min; *p* = 0.33, *t* = 0.99, df = 18, *F*-value = 1.035) (Figure 3D).

### *Glra1* Mutant Mice Are Not Anxious to Cross Open Spaces

**FIGURE 4:**
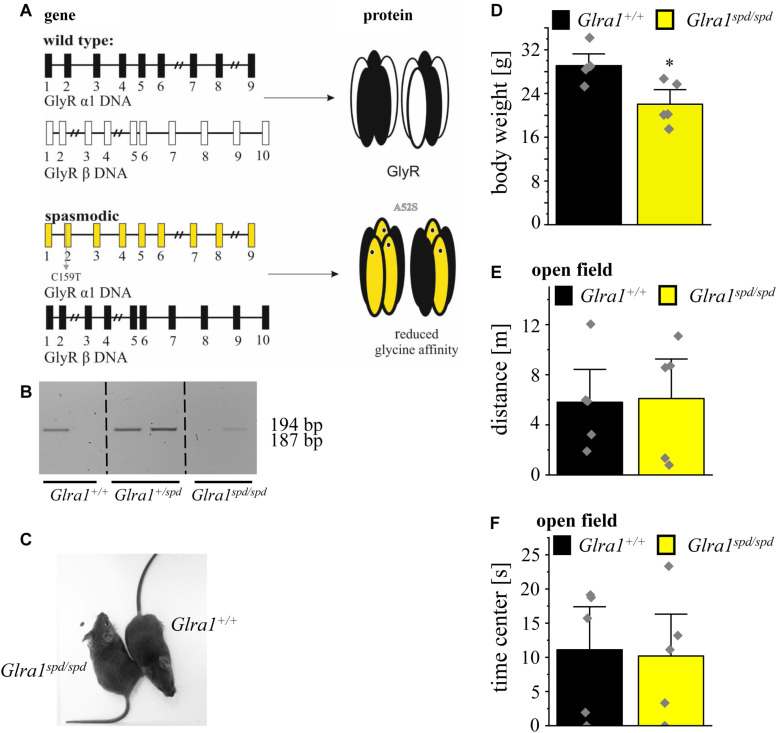
Homozygous *spasmodic* mice show no anxiety-like behavior. **(A)** Schematic overview of *Glra1* (9 exons) and *Glrb* (10 exons) genes in *spasmodic* mice. Upper part shows the wild type situation (black and white), lower part depicts the situation in *spasmodic* mice (yellow and black). Left column depicts changes at DNA level, note the base pair exchange C159T in exon 2 of *Glra1* (yellow) which leads to a missense mutation A52S at the protein level. Right column: Protein level of GlyR with α1^*A52S*^ marked by a black dot within the GlyR α1 subunit (yellow). **(B)** Genotyping of *Glra1^+/+^*, *Glra1^+/spd^*, and *Glra1*^*spd/spd*^ mice. Wild type animals show a single 194 bp band, heterozygous animals show two amplimers (194 and 187 bp), homozygous *spasmodic* mice display a band at 187 bp. **(C)** Images of *Glra1^+/+^* and *Glra1^*spd*/*spd*^* animals are shown. **(D)** Body weight of *Glra1^*spd*/spd^* mice (*n* = 5, yellow bar) and *Glra1^+/+^* mice (*n* = 5, black bar). Level of significance refers to ^∗^*p* < 0.05. **(E)** Traveled distance within an open field arena of *Glra1^*spd*/spd^* mice (*n* = 5, yellow bar) and *Glra1^+/+^* mice (*n* = 5, black bar). **(F)** Time spent in the center of the open field documented for *Glra1^*spd*/spd^* mice (*n* = 5, yellow bar) and *Glra1^+/+^* mice (*n* = 5, black bar). Data points are shown as gray color squares.

To study if the *Glra1* gene has an impact on anxiety-related behavior, we used homozygous *spasmodic* mice. *Spasmodic* is a mouse line carrying a spontaneous mutation A52S in the *Glra1* gene resulting in hyperekplexia (Figure 4A) due to lower affinity to the agonist glycine (Saul et al., 1994). It has been reported from humans carrying a *GLRA1* mutation and thus suffering from hyperekplexia that the affected patients are anxious to fall upon exposure to unexpected noise or tactile stimuli (Andermann et al., 1980).

Following genotyping (Figure 4B), homozygous *spasmodic* mice were investigated for changes in their outer appearance e.g., quality of fur (color and thickness). Except provoking a startle reaction, homozygous *spasmodic* mice cannot be distinguished from wild type littermates (Figure 4C). In addition, no differences in body weight were detectable. Homozygous *spasmodic* mice exhibited less body weight but had no obvious constraints in motor behavior (*Glra1^+/+^ n* = 5, 29.1 ± 1.4 g; *Glra1*^*spd/spd*^ *n* = 5, 22.4 ± 1.8 g; *p* = 0.0148, *t* = 3.095, df = 8, *F*-value = 1.526) (Figure 4D).

To exhibit if a mutation in the *Glra1* gene interferes with anxiety-related behavior, homozygous *spasmodic* mice were tested in the open field paradigm. Neither the distance traveled (*Glra1*^*spd/spd*^ *n* = 5, 6.1 ± 2.1 m; *Glra1^+/+^ n* = 5, 5.8 ± 1.8 m; *p* = 0.92, *t* = 0.1088, df = 8, *F*-value = 1.455) (Figure 4E) within the open field arena nor the time spent within the center (*Glra1*^*spd/spd*^ *n* = 5, *Glra1^+/+^ n* = 5, 11.1 ± 4.2 s; 10.2 ± 4.1 s; *p* = 0.87, *t* = 0.15, df = 8, *F*-value = 1.054) (Figure 4F) of the open field differed between wild type and homozygous *Glra1*^*spd/spd*^ littermates. Thus, a mutation in the *Glra1* gene had no impact on anxiety-related behavior at least not in the open field test investigated here.

### *Spasmodic* Mice Show Extensive Startle Reactions With Fear-Related Behavior During Startle

We further focused on the startle reaction paradigm in mice, a typical symptom in hyperekplexia in humans and in rodents. Moreover, it was previously shown that probands with polymorphisms within the *GLRB* gene show an increased startle behavior (Deckert et al., 2017).

**FIGURE 5:**
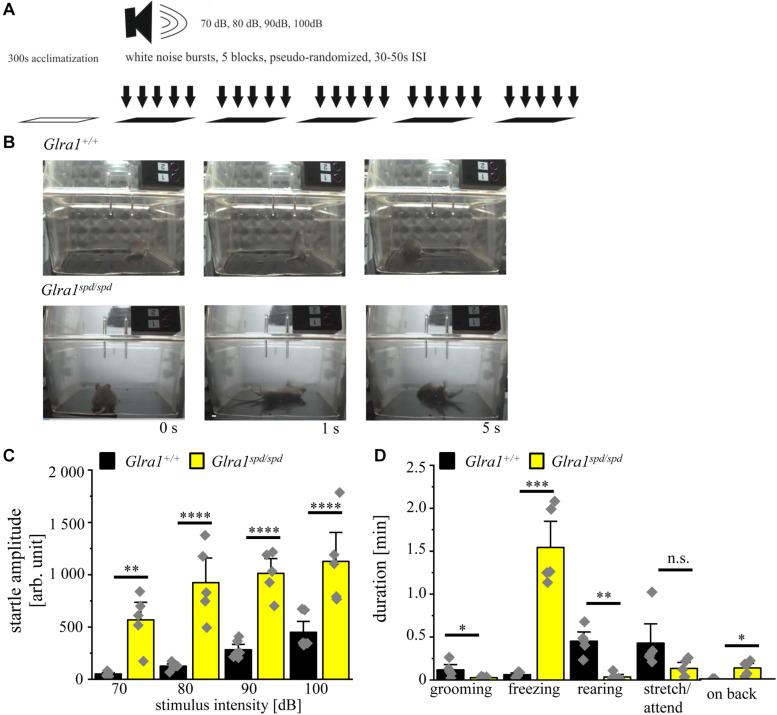
Homozygous *spasmodic* mice show extensive startle reactions. **(A)** Schematic overview of the conducted startle test. After 300 s of acclimatization, five blocks with white noise bursts of 70–100 dB in pseudo-randomized order were applied with 30–50 s pause between blocks. **(B)** Representative images of *Glra1^+/+^* and *Glra1*^*spd/spd*^ animals during startle test. Note that *Glra1*^*spd/spd*^ fall on their back immediately after the tone due to evoked startle responses. **(C)** Startle amplitude of *Glra1*^*spd/spd*^ (*n* = 5, yellow bars) and *Glra1^+/+^* (*n* = 6, black bars) animals during behavioral startle test are depicted with increasing stimulus intensities (70–100 dB). **(D)** Times spent grooming, freezing, rearing, stretch/attend, and laying on back of *Glra1*^*spd/spd*^ (*n* = 5, yellow bars) and *Glra1^+/+^* (*n* = 6, black bars) animals were tracked during the startle test (1110 s). Level of significance refer to ^∗^*p* < 0.05, ^∗∗^*p* < 0.01, ^∗∗∗^*p* < 0.001, ^****^p < 0.0001. Gray squares refer to single data points.

A schematic overview about the test paradigms to evoke a startle response by increased intensities of acoustic noises is shown in Figure 5A. During the test, mice were exposed to increasing sound pressure levels of 70–100 dB in five blocks with pseudo-randomized order and the startle reaction was analyzed with the help of a piezo sensitive platform to measure the startle amplitude. An increase in startle response was visible for homozygous *spasmodic* mice exhibiting a sound induced startle attack, leading to falls on their backs with prolonged righting time (Figure 5B). In contrast, wild type mice did not fall on their backs and displayed only minor startle reactions. Analyzing the startle amplitude of homozygous *Glra1*^*spd/spd*^ mice, a significant increase in the startle responses was detected with increasing stimulus intensities (repetitive ANOVA (genotype × stimulus intensity) *F*(3,27) = 1.728; *p* = 0.1850; *Glra1*^*spd/spd*^ *n* = 5, 70 dB: 568 ± 125; 80 dB: 924 ± 176; 90 dB: 1013 ± 105; 100 dB: 1127 ± 206; *Glra1^+/+^ n* = 5, 70 dB: 52 ± 8; 80 dB: 126 ± 14; 90 dB: 283 ± 34; 100 dB: 450 ± 69; 70 dB: *p* = 0.002, *t* = 3.824; 80 dB: *p* < 0.0001, *t* = 5.91; 90 dB: *p* < 0.0001, *t* = 5.407; 100 dB: *p* < 0.0001, *t* = 5.019; Bonferroni *post hoc* test) (Figure 5C).

We further investigated fear-related behavior during the startle test. The time spent grooming, freezing, rearing, stretch/attend and time spent on back were analyzed. Homozygous *spasmodic* mice spent less time grooming and rearing, but spent much more time freezing and on their backs (*Glra1*^*spd/spd*^ *n* = 5, grooming: 0.03 ± 0.01 min *p* = 0.056, *t* = 2.23, df = 8, *F*-value = 30.83, freezing: 1.5 ± 0.2 min, *p* < 0.0001, *t* = 7.3, df = 8, *F*-value = 242, rearing: 0.03 ± 0.02 min, *p* = 0.0005, *t* = 5.636, df = 8, *F*-value = 14, stretch/attend: 0.13 ± 0.05 min, *p* = 0.09, *t* = 1.874, df = 8, *F*-value = 10.46, on back: 0.13 ± 0.04 min, *p* = 0.0066, *t* = 3.640, df = 8, *F*-value = invinity; *Glra1^+/+^ n* = 5, grooming: 0.12 ± 0.04 min, freezing: 0.06 ± 0.01 min, rearing: 0.45 ± 0.07 min, stretch/attend: 0.43 ± 0.15 min, on back: 0 ± 0 min) (Figure 5D). In summary, homozygous *spasmodic* mice showed an enhanced startle reaction with multiple significant alterations in fear-related behavior during the startle test.

### Heterozygous *Spastic* Mice Lack Enhanced Startle Responses

**FIGURE 6:**
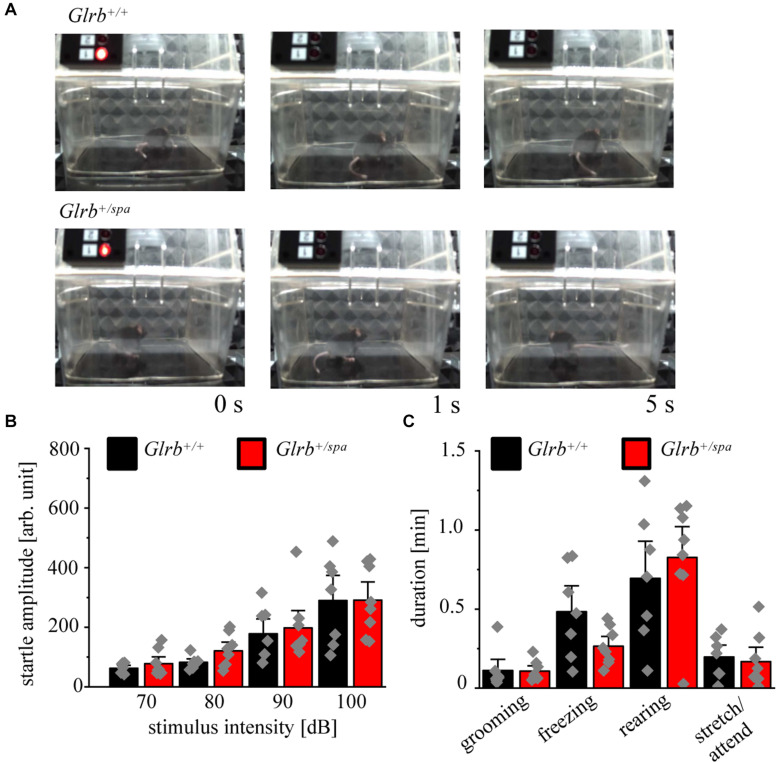
Heterozygous *spastic* mice lack enhanced startle responses. **(A)** Representative images of *Glrb^+/spa^* (*n* = 8, red bars) and *Glrb^+/+^* (*n* = 7, black bars) animals during behavioral startle test. **(B)** Startle amplitudes of *Glrb^+/spa^* and *Glrb^+/+^* animals during behavioral startle test using increased stimulus intensities (70–100 dB). **(C)** Times spent grooming, freezing, rearing, and stretch/attend of *Glrb^+/spa^* (*n* = 8, red bars) and *Glrb^+/+^* (*n* = 7, black bars) animals during the startle test (time 1110 s). Data points are shown as gray color squares.

The established startle paradigm was used to study the startle responses in heterozygous *spastic* mice. Here, no visible changes in the startle response were apparent in heterozygous *spastic* mice compared to controls. Note that heterozygous *spastic* mice did not fall on their backs as homozygous *spasmodic* mice (Figure 6A).

The startle amplitudes estimated in *spastic* mice were unaltered between heterozygous *spastic* animals and control wild type littermates [repetitive ANOVA (genotype × stimulus intensity) *F*(3,39) = 0.1642; *p* = 0.9198; *Glrb^+/spa^ n* = 8, 70 dB: 78 ± 15; 80 dB: 121 ± 19, *p* = 0.1; 90 dB: 198 ± 39; 100 dB: 115 ± 41; *Glrb^+/+^ n* = 7, 70 dB: 67 ± 8.6, 80 dB: 90 ± 10, 90 dB: 199 ± 41, 100 dB: 1499 ± 230; *p* > 0.9999 for all conditions 70, 80, 90, and 100 dB with *t* = 0.3448 (70 dB); *t* = 0.8568 (80 dB); *t* = 0.4319 (90 dB), *t* = 0.02958 (100 dB); Bonferroni *post hoc* test] (Figure 6B). We also documented changes in fear-related behaviors, such as grooming, freezing, rearing and stretch/attend. No significant changes between both genotypes were detectable (*Glrb^+/sp*a*^ n* = 8, grooming: 0.11 ± 0.02 min, *p* = 0.9, *t* = 0.08, df = 13, *F*-value = 3.940; freezing: 0.27 ± 0.04 min, *p* = 0.07, *t* = 1.972, df = 13, *F*-value = 6.0; rearing: 0.83 ± 0.13 min, *p* = 0.52, *t* = 0.65, df = 13, *F*-value = 1.281; stretch/attend: 0.17 ± 0.06 min, *p* = 0.72, *t* = 0.36, df = 13, *F*-value = 1.631; *Glrb^+/+^ n* = 7, grooming: 0.11 ± 0.05 min, freezing: 0.48 ± 0.12 min, rearing: 0.69 ± 0.16 min, stretch/attend: 0.2 ± 0.05 min) (Figure 6C). Thus, heterozygous *spastic* mice do not show an increase in fear-related behaviors in comparison to wild type littermates.

### Pavlovian Fear Conditioning Behavior of *Glrb^+/+^* and *Glrb^+/spa^* Animals

**FIGURE 7:**
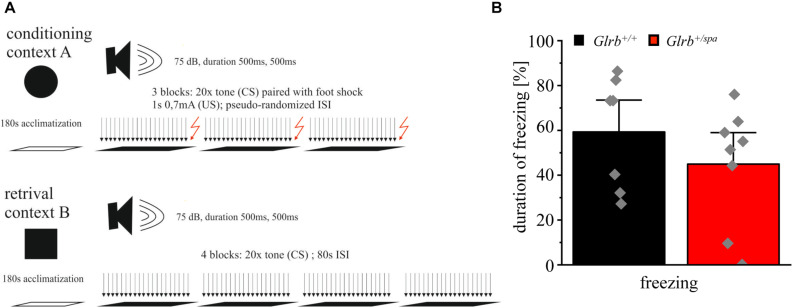
Wild type and *spastic* mice show similar contextual fear behavior. **(A)** Schematic overview of the conducted fear conditioning test: Context A shows 180 s of acclimatization followed by three blocks of 20 tones (CS) followed by a foot shock (US) of 1 s and 0.7 mA. Inter-stimulus-intervals (ISIs) are pseudo-randomized. Context B shows the same paradigm without applying foot shocks (US) after each block (four blocks). **(B)** Duration of freezing behavior of *Glrb^+/spa^* (*n* = 8; red bar) and *Glrb^+/+^* (*n* = 7; black bar) animals shown as the percentage of total time spent freezing with respect to total duration of the fear conditioning paradigm is presented as mean ± S.E.M. Gray squares refer to single data points obtained for one animal.

To further investigate the startle behavior of heterozygous *spastic* mice, a Pavlovian fear conditioning test was performed (Figure 7A). Pavlovian fear conditioning in mice is a typical paradigm to test for associative learning and memory processing (Johansen et al., 2011; LeDoux, 2014; Tovote et al., 2015). Plasticity defects in amygdala and brain stem circuits are known to interfere with the conditioning of defensive behavior to an auditory cue (tone) (Tovote et al., 2015). In this test, the freezing duration was unchanged in heterozygous *spastic* compared to control animals (*Glrb^+/spa^ n* = 8, freezing 45 ± 9.4%; *Glrb^+/+^ n* = 7, freezing: 59 ± 9.5%; *p* = 0.3, *t* = 1.077, df = 13, *F*-value = 1.445) (Figure 7B). In summary, our data demonstrated that heterozygous *spastic* mice do not differ in auditory fear from wild type controls.

## Discussion

Previous studies linked the *GLRB* gene to anxiety disorders, like panic disorder and agoraphobia. Panic disorders are characterized by a rapid rise in anxiety, accompanied by accelerated heart rate, shortness of breath, and losing control upon fear. Emotions and behavioral responses in panic disorders are encoded by a complex, not fully understood, interplay of different neural circuits in the brain (Godemann et al., 2005; Hamm et al., 2016). In humans carrying single non-coding nucleotide *GLRB* variants, increased startle reflexes combined with an avoidance of open spaces were the main behavioral characteristics (Deckert et al., 2017; Lueken et al., 2017).

The present study investigated *spastic* mice carrying a *Glrb* mutation and *spasmodic* mice with a *Glra1* mutation to study anxiety- and fear-related behavior in rodents. Although both mutant mouse lines have different genetic backgrounds (*spastic* C57/BL6; *spasmodic* C3H), C57/BL6 and C3H wild type mice show similar responses to acoustic stimuli. Moreover, the C57/BL6 strain is often used to study fear and anxiety and displays less anxiety-related behavior relative to e.g., 129 substrains (Crawley et al., 1997; Rodgers et al., 2002).

*Spastic* mice display various GlyR β transcripts generated by aberrant splicing as a consequence of a LINE-1 element insertion into intron 6 of the *Glrb* gene. We have observed no alterations in body weight, traveled distances, and pain sensitization in heterozygous *spastic* animals (display around 70–80% full-length GlyR β compared to wild type control animals as a prerequisite to study anxiety-related behavior.

In contrast to the rodent model with reduced GlyR β levels, the rs7688285 variant of the human *GLRB* gene increased *GLRB* transcript expression especially in the midbrain, while expression levels in the forebrain and amygdala were unaffected (Deckert et al., 2017). From genetic *GLRA1* and *GLRB* variants leading to motor deficits, increased GlyR β expression has not yet been reported. Reduced GlyR expression level or loss of function mutations, however, generate impaired glycinergic signaling, which accounts for muscle stiffness, and increased startle reactions upon sudden tactile or acoustic stimuli. This can even lead to episodes of apnea, when breathing is affected (Manzke et al., 2010). Existing genetic gain of function variants demonstrated increased intracellular chloride level resulting in reduced GlyR cluster numbers and sizes in mature spinal cord neurons (Schwale et al., 2016; Zhang et al., 2016). Increased GlyR β expression as present in *GLRB* risk allele carriers might underlie similar processes resulting in excitation/inhibition imbalances finally explaining the observed general higher defensive reactivity. Therefore, the differences in GlyR β levels in heterozygous *spastic* animals investigated here and in human *GLRB* risk allele carriers might lead to differences in glycinergic signaling.

GlyRs of the α1β or α3β subtypes are mainly expressed in the adult brainstem and spinal cord mediating controlled motor coordination by recurrent inhibition of motoneurons (Malosio et al., 1991; Harvey et al., 2004; Schaefer et al., 2012; Liu and Wong-Riley, 2013). However, GlyR α2 and β transcripts forming homomeric α2 and heteromeric α2β receptors were also identified in the midbrain, cortex, thalamus, lateral amygdala, hippocampus, and cerebellum (Malosio et al., 1991; Waldvogel et al., 2007). GlyR α2 also regulates cortical neurogenesis in the forebrain and promotes interneuron migration (Avila et al., 2013; Morelli et al., 2017). In the adult, α2β receptors are the major subtype in the prefrontal cortex, striatum, and hippocampus regulating neuronal excitability (Jonsson et al., 2012; McCracken et al., 2017). The high abundance of GlyR β in these brain regions might hint to further so far unknown functions of the GlyR β subunit.

Human *GLRB* carriers showed strong activation of the defensive system of the brain specifically in the thalamus and insula. In the early phase of fear conditioning also inverse effects have been observed indicating impaired fear inhibitory learning (Deckert et al., 2017; Lueken et al., 2017). The heterozygous *spastic* mice investigated in this study displayed no significant differences in the EPM or in the light-dark test. This contrasts previous findings, which revealed an avoidance of open spaces in heterozygous *spastic* mice similar to findings from human *GLRB* risk alleles (Deckert et al., 2017). As adult GlyRs are always composed of α and β subunits, we analyzed *spasmodic* mice carrying a *Glra1* mutation in the open field test. Homozygous *spasmodic* mice were, however, indistinguishable from wild type control animals. This result was not unexpected as the GlyR α1 subunit is almost absent from brain regions involved in fear-related behavior, e.g., forebrain, amygdala, thalamus, and hippocampus (Malosio et al., 1991). While human *GLRB* risk allele carriers show anxiety symptoms, they do not suffer from pathological anxiety (Lueken et al., 2017).

A modulation of the startle reflex was identified in human *GLRB* single non-coding nucleotide polymorphism carriers (Deckert et al., 2017; Lueken et al., 2017). Early case reports of human genetic GlyR variants suffering from hyperekplexia mentioned startle attacks evoked by surprise, stress and fear (Kirstein and Silfverskiold, 1958). The acoustic startle response (ASR) to sudden intense stimuli involves neural circuits in the lower brainstem with the caudal pontine reticular nucleus (PnC) as a key element besides the cochlear root neuron and the motoneurons (Koch, 1999; Lang et al., 2000). Genetic mouse models for hyperekplexia show extreme startle responses upon unexpected noise or touch. *Spasmodic* mice displayed significant increases in the startle amplitudes concomitant to enhanced noise intensity. Hence, the *Glra1* defect in *spasmodic* mice cannot be compensated by other α subunits or the β subunit expressed in brainstem. In contrast, heterozygous *spastic* mice do not show enhanced startle responses arguing that the presence of around 70% of full-length GlyR β does not impair the signaling cascade from the cochlear neurons via PnC neurons toward the output motoneurons in the brainstem. Our data are in line with observations from heterozygous *spasmodic* mice and recessive hyperekplexia mutations in humans indicating that one mutated allele (50% GlyR α or β expression) does not disturb glycinergic inhibition with a typical startle phenotype thereof (Plappert et al., 2001; Chung et al., 2010; Schaefer et al., 2015).

The startle reflex is also used to study fear and anxiety states (Davis et al., 2010). Fear states driven by external threat or internal association are defined by defense system activation and its reflexive autonomic (heart beats) and somatic (startle reflex) output (Lang et al., 2000). The defense system, which involves the same brain structures in humans and rodents, includes a subcortical circuit with the central amygdala and outputs to the periaqueductal gray (PAG) and PnC organizing the mode of defense (Fanselow, 1994; Fendt and Fanselow, 1999). Hence, the circuit of the defensive system converges at the level of the PnC with the ASR signaling pathway. Brain nuclei involved in both pathways express GlyR β (Malosio et al., 1991). The defensive phenotype is also characterized by a freezing paradigm. Freezing is described first as crouching, meaning complete absence of movement except for that associated with respiration and tense body posture. Tense body posture includes increased muscle tone (is a third characteristic) (Hagenaars et al., 2014). Interestingly, extreme freezing during the startle paradigm was observed for *spasmodic* mice. Moreover, *spasmodic* mice spent less time grooming and rearing but increased time on their back, in sum showed multiple significant alterations in fear-related behavior. Hence, the glycinergic system might be able to modulate fear conditions.

Learned fear induced by Pavlovian fear conditioning contributes to an enhancement of the ASR. Fear (threat) conditioning combines a conditioned stimulus (CS, such as a tone) with an aversive stimulus (US, like a foot shock). Relay neurons between the amygdala and the PnC such as the PAG contribute to ASR sensitization (Hagenaars et al., 2014). Using classical fear conditioning, the freezing duration was unchanged in heterozygous *spastic* mice compared to wild type controls. As has been pointed out before, the small reduction of the full-length GlyR β protein as present in *spastic* mice does not lead to impaired glycinergic signal transduction. The presence of GlyR β in neural circuits involved in fear-related behavior from the amygdala to the PnC and finally to the output motoneurons suggests that GlyR signaling and most probably the neurotransmitter glycine might display a modulatory function in these circuits. So far, GABA has been demonstrated to inhibit defensive behavior in the amygdala and the PAG (Tovote et al., 2015, 2016). However, mixed GABAergic/glycinergic synapses that have been described in the brainstem and spinal cord might also exist in other brain regions.

## Conclusion

In conclusion, our data showed only subtle differences in fear and anxiety-like phenotypes in *spastic* compared to wild type littermates. While functional alteration of the GlyR α1 in homozygous *spasmodic* mice led to enhanced anxiety-like behavior during the acoustic startle test and to increased startle reactivity, the reduction of GlyR β level in heterozygous *spastic* mice had no effect on anxiety-related behavior.

## Data Availability Statement

The datasets generated for this study are available on request to the corresponding author.

## Ethics Statement

The animal study was reviewed and approved by the local veterinary authority (Veterinäramt der Stadt Würzburg) and Committee on the Ethics of Animal Experiments, i.e., Regierung von Unterfranken, Würzburg (License numbers 55.2-2531.01-09/14 and 55.2.2-2532.2-536-28).

## Author Contributions

CV, JD, RB, NS, BW, and PT participated in research design. CV, NS, JS-G, and CC conducted the experiments. CV, NS, JS-G, CC, and PT performed the data analysis. CV and NS wrote the manuscript. All authors contributed to the article and approved the submitted version.

## Conflict of Interest

The authors declare that the research was conducted in the absence of any commercial or financial relationships that could be construed as a potential conflict of interest.
